# Challenging school-related situations faced by students with dyslexia: a scoping review

**DOI:** 10.1007/s11881-025-00356-9

**Published:** 2025-12-12

**Authors:** Julia Mori, Katharina Prinz, Ursula Fischer, Liliana Tönnissen, Claudia Hofmann, Jennifer Plank, Dennis Hövel, Alfred Schabmann, Barbara Gasteiger-Klicpera, Barbara M. Schmidt

**Affiliations:** 1 Institute for Educational Support for Behaviour, Social-Emotional, and Psychomotor Development, University of Teacher Education in Special Needs (HfH), Schaffhauserstrasse 239, Zurich, 8050 Switzerland; 2 Department of Education Research and Teacher Education, Inclusive Education Unit, University of Graz, Merangasse 70/II, Graz, 8010 Austria; 3 Center for Education and Training, University of Teacher Education in Special Needs (HfH), Schaffhauserstrasse 239, Zurich, 8050 Switzerland; 4 Institute for Learning under Difficult Conditions, University of Teacher Education in Special Needs (HfH), Schaffhauserstrasse 239, Zurich, 8050 Switzerland; 5 Faculty of Human Sciences, Department of Rehabilitation and Special Education, University of Cologne, Klosterstrasse 79b, 50931 Cologne, Germany; 6 Research Center for Inclusive Education (CERI), University of Graz, Strassoldogasse 10/II, Graz, 8010 Austria

**Keywords:** Dyslexia, School context, School-related challenges, Scoping review

## Abstract

Children and young people with dyslexia face a variety of challenges in everyday school life, which can affect their intellectual and emotional development. While the potential consequences of dyslexia are well-documented, little is known about the specific challenging situations in the school context these children face. This scoping review addresses this gap by systematically identifying and categorising school-related stressors experienced by students with dyslexia. A literature search between 2000 and 2025 was conducted via EBSCOhost across five databases in the fields of education and psychology. A total of 30 peer-reviewed studies were included for full-text analysis. The majority of studies (*n* = 23) were qualitative, complemented by quantitative (*n =* 4) and mixed-method studies (*n =* 3). All participants had a formal diagnosis of dyslexia and described their school-related experiences either currently or retrospectively. The reported school-related challenging situations were assigned to two main categories: social environment and student-related stressors. Many typical school routines—such as reading aloud in front of the class, peer comparisons, or public error correction—emerged as particularly distressing. These practices are rarely questioned but can lead to humiliation, anxiety, and fear of failure for students with dyslexia. Recognising the subjective experiences of students with dyslexia offers valuable insights for developing more inclusive educational practices and targeted socio-emotional support.

**Supplementary Information:**

The online version contains supplementary material available at https://doi.org/10.1007/s11881-025-00356-9.

## Introduction

Difficulties in reading and writing—including developmental dyslexia—are linked not only to learning challenges but also to emotional and behavioral difficulties that can negatively shape a child’s development and school experience. Research suggests that around 40% to 60% of children and adolescents with dyslexia develop psychological symptoms, with some of the core symptoms persisting into adulthood (Schulte-Körne, 2010; Undheim, 2003). This rate is significantly higher than global prevalence estimates for psychological disorders among 5–17-year-olds (3% to 16%, depending on the disorder; Erskine et al., 2017), underscoring the heightened risk in samples with dyslexia. Dyslexia is frequently associated with a range of externalizing (e.g., aggression) and internalizing (e.g., anxiety) behavioral difficulties (e.g., Roberts et al., 2020; Turunen et al., 2018; Visser et al., 2020; Zuppardo et al., 2021).

More broadly, meta-analytic evidence indicates that struggling readers—irrespective of formal diagnosis—show elevated rates of internalizing difficulties compared with peers (Francis et al., 2019). Children and adolescents with dyslexia and/or other learning difficulties have to deal with perceived threats to their self-esteem within the school environment and report feeling stressed, confused, annoyed, and embarrassed by their learning challenges (Wilmot et al., 2023). Learning disabilities, including developmental dyslexia, are associated with poorer mental-health outcomes and, in turn, with reduced opportunities across schooling, relationships, and employment (Livingston et al., 2018). Against this backdrop, although we focus on the lived school experiences of students with dyslexia, many practical implications likely extend to the wider population of students with persistent literacy/learning challenges. Much is already known about the prevalence of emotional and behavioral difficulties of students with dyslexia and their potential long-term consequences. However, what remains unclear is which specific situations students with dyslexia experience as particularly challenging and how these situations manifest within the school context. This scoping review addresses this gap by systematically identifying and categorizing such situations.

## Conceptual framework of the current study

School-related social and emotional challenges faced by students with dyslexia can be discussed in relation to three well-established frameworks: the bioecological model of human development (Bronfenbrenner & Morris, 2007; Rosa & Tudge, 2013), the person–environment fit theory (Hunt, 1975; Moos, 1987), and the social model of disability (Oliver, 1996). The bioecological and person–environment fit frameworks share the premise that human development results from continuous interactions between individuals and their environments. According to the bioecological model, these interactions occur within multiple, interconnected systems ranging from immediate settings such as the family and classroom (microsystem) to broader social, cultural, and institutional contexts (macrosystem). Students’ development is therefore shaped by both proximal processes—such as teacher support and peer relationships—and distal influences, including school policies and societal attitudes. Building on this view, Heimlich (2022) emphasizes that the entire life and concrete learning situations of children and adolescents must be considered when analyzing development and educational participation, as learning and development are embedded in lived experiences, material and social contexts, and subjective perceptions.

Similarly, the person–environment fit theory emphasizes that the degree of congruence between students’ characteristics and their environment determines how well they adjust and thrive. The school environment thus plays a central role in how students with learning difficulties experience school and whether they feel they belong. When this fit is inadequate—when instructional demands, social expectations, or school climates do not align with students’ needs—students are more likely to experience maladjustment and disengagement (Eccles et al., 1993; Finn, 1989; Rovai et al., 2005). Conversely, environments that provide responsive teaching, inclusive social climates, and appropriate support systems promote more positive academic, emotional, and social outcomes (Way et al., 2007; Eccles & Midgley, 1989).

From a complementary perspective, the social model of disability views disability as socially constructed (Oliver, 1996) and argues that people are not disabled by internal factors but rather by a society that fails to accommodate their differences (Wilmot et al., 2023). Accordingly, difficulties experienced by students with dyslexia reflect environmental barriers, cultural attitudes, and social biases that shape their participation in school life, and not merely the result of their impairments (Andrews et al., 2022). These social dynamics intersect with labeling: diagnostic labels shape how people are perceived and treated within their social contexts (Link & Phelan, 2001). In educational settings, labels such as “dyslexia” can influence teacher expectations, peer attitudes, and students’ self-concept (Ho, 2004; Riddick, 2000). From a social-model perspective, the consequences of labeling stem less from the label itself than from the cultural meanings and institutional responses attached to it. Deficit-oriented framings can fuel stigma and exclusion, whereas inclusive, strengths-based practices enable understanding, identity validation, and access to resources (Abbey & Valsiner, 2005; Blajeski et al., 2022; Sims et al., 2021). Acknowledging labeling effects therefore adds nuance to the social construction of disability and underscores the need for culturally sensitive communication and systemic change in how learning differences are represented in schools. Considering these perspectives together, research investigating ways to improve the mental health of students with dyslexia needs to look beyond within-child factors and consider the broader “person–environment fit” (Mandy, 2019, p. 1880).

Despite growing awareness of the behavioral difficulties associated with dyslexia, school-based support often focuses narrowly on reading and spelling, while social and emotional challenges are typically left to child and adolescent psychiatric services (Hendren et al., 2018). Particularly concerning is that school-based reading interventions and structural arrangements like ability grouping (Lithari, 2019) may unintentionally stigmatize them (Deacon et al., 2020). Studies investigating the social and emotional experiences of students with dyslexia consistently emphasize the possible negative impact of classroom dynamics, particularly of interactions with teachers and peers. The ways in which teachers organize instruction and respond to learning difficulties can either diminish or enhance students’ socio-emotional adjustment. Students’ own awareness of their dyslexia diagnosis also plays a complex role in school life and can elicit mixed emotions—ranging from relief at having an explanation for academic difficulties to long-lasting emotional burdens and a persistent sense of limitation (Deacon et al., 2020; Gibson & Kendall, 2010). Beyond the classroom, homework emerges as another significant source of stress for students with dyslexia. Reading-related tasks at home often require a disproportionate amount of time and effort, affecting not only the child but the entire family life (Xiao et al., 2022). From the child’s perspective, the additional time spent on homework reduces opportunities for leisure, extracurricular activities, and peer interactions (Leitão et al., 2017; Rowan, 2010; Xiao et al., 2022). Taken together, these findings illustrate a complex web of social and emotional school-related stressors that affect students with dyslexia within a broader landscape of literacy-related mental-health risk.

### The present review

The aim of this scoping review is to provide an overview of which school-related situations students with dyslexia perceive as challenging. The review analyzes studies in which either children and adolescents diagnosed with dyslexia, their parents, and/or their teachers reflect on their challenging school experiences (either currently or retrospectively). Scoping reviews are an increasingly common approach to evidence synthesis and are designed to identify and map the breadth and nature of relevant evidence that meets pre-determined inclusion criteria regarding the topic (Peters et al., 2021). They are particularly appropriate when the aim is to present and describe relevant characteristics of included sources of evidence rather than to estimate pooled effects (Tricco et al., 2018). Scoping reviews systematically identify relevant studies available on a given topic, with the aim of addressing stated objectives and review questions while highlighting central concepts, data, and evidence gaps (McGowan et al., 2020). In line with these aims, we used a scoping review to chart school-related situations perceived as challenging by students with dyslexia across diverse contexts, and to surface recurrent themes that can inform more inclusive educational practice, future research, and comprehensive support programs that take the full range of experiences into account. Thereby, this review seeks to answer the research question: *Which school-related situations are perceived as challenging by students diagnosed with dyslexia?*

## Method

To address our research question, we applied the Preferred Reporting Items for Systematic reviews and Meta-Analyses extension for Scoping Reviews (PRISMA-ScR) guidelines (Tricco et al., 2018). Unlike systematic reviews, which follow a rigid protocol, scoping reviews allow for broader research questions and an iterative, reflective process. Given the limited synthesis of school-related situations experienced as stressful by children with dyslexia, this approach enables the identification of research gaps and the mapping of existing evidence (Peters et al., 2015).

### Research strategy

The literature search was conducted in November 2021 and March 2025 via EBSCOhost in the databases ERIC, APA PsychInfo, PSYNDEX, MEDLINE und Education Source. The search strings used for the literature search can be seen in Table 1. To ensure that we identified relevant empirical research articles, we discussed and revised the search terms and searched for the terms in the abstracts.

**Table 1:** Overview of the search strings

Component 1	Component 2 (AND)	Component 3 (AND)
“struggling reader*” OR dyslexia OR “reading disorder*” OR “reading difficult*” OR “reading problem*” OR “writing disorder*” OR “writing difficult*” OR “writing problem*”	“mental health” OR frustrat* OR emotional OR conflict* OR external* OR internal* OR coping OR cope OR undercontrol* OR overcontrol* OR “task avoidan*” OR “behavi#r* problem*” OR “behavi#r* disorder*” OR “school experience*”	school* OR lesson* OR teacher* OR pupil* OR student* OR class* OR peer*
Component 4 (NOT)		
stroke OR “cerebrovascular accident” OR cva OR aphasia OR agraphia OR alexia OR asd OR “autism spectrum disorder*” OR autism OR adhd OR “attention deficit hyperactivity disorder” OR “attention deficit-hyperactivity disorder” OR “covid-19” OR coronavirus OR lockdown OR pandemic		

## Study selection

**Fig. 1:**
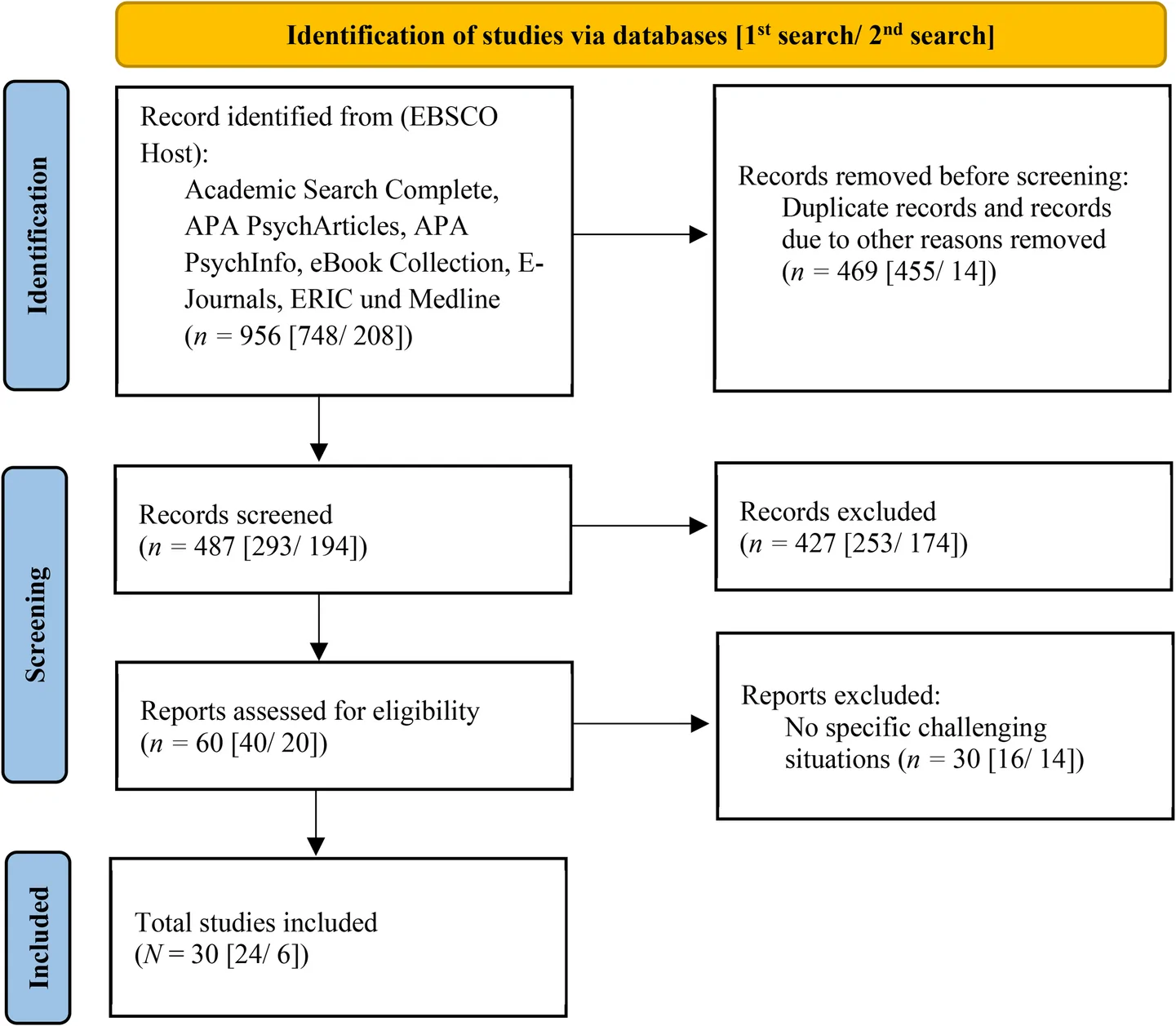
PRISMA flow diagram. (adapted from Page et al., 2021)

Study selection was guided by six predefined criteria (see Table 2). The *first* database search (articles published between 01/2000 and 11/2021) conducted in November 2021 resulted in 748 studies. After removing duplicates, 293 abstracts were independently screened by three researchers, ensuring that each abstract was assessed by two reviewers. A total of 40 studies were identified as referencing specific challenging situations encountered by students with dyslexia. In cases of agreement between reviewers, the article was either included or excluded accordingly. In cases of disagreement, a third researcher made the final decision. Ultimately, 24 publications were selected for in-depth analysis (see PRISMA diagram, Fig. 1), as their full texts provided detailed descriptions of concrete challenges faced by students with dyslexia. The *second* database search (articles published between 11/2021 and 03/2025) conducted in March 2025 followed the same procedure and yielded 208 studies. After the independent screening of 194 abstracts by three researchers, 20 studies were identified as relevant, and six publications were selected for in-depth analysis (see PRISMA diagram, Fig. 1). Thus, the first and second database searches resulted in a total of 30 publications that were included in the full-text analysis.

**Table 2:** Inclusion and exclusion criteria

Inclusion criteria	Exclusion criteria
Concrete challenging situations of students with dyslexia in the school context	No specific challenging situations (e.g., focus on the association between dyslexia and behavior)
Students (or parents/caregivers, teachers, if they refer to the student’s situation) report from a current or retrospective perspective	Wrong target group (e.g., parents/caregivers or teachers’ reports)
Diagnosis of dyslexia	Main diagnosis other than dyslexia
Students report on their time in regular schooling	Focus is on an educational institution other than regular school (e.g., special education school)
Qualitative/quantitative empirical studies (peer-reviewed)	Grey literature, theses, theoretical journal articles
Published since 2000	Published before 2000
Published in German or English	Published in a language other than German or English

### Data analysis

We analyzed the data on challenging situations encountered by students with dyslexia using MAXQDA 2024 (VERBI Software, 2021). Codes were assigned using a combined deductive and inductive coding system (Kuckartz, 2018; Rädiker & Kuckartz, 2019). Based on the analysis of the challenging situations, two main categories were identified: social environment and student-related stressors. An overview of the main categories and their corresponding subcategories is provided in Table 3. The coding manual provides descriptions of each (sub)category, accompanied by illustrative anchor examples (see Supplementary Table S1). Prior to the coding process, three researchers participated in a coding training session. To assess inter-rater reliability (IRR), 15 articles were double-coded in the first research phase (November 2021), yielding Cohen’s kappa values ranging from κ = 0.91 to κ = 0.96. In the second research phase (March 2025), all six articles were double-coded, resulting in an IRR of κ = 0.90.

**Table 3:** Overview of the main categories and subcategories

Main category	Subcategory
Social environment	Negative perceptions of parents/guardians (D) Conflicts with parents/guardians/siblings (D) Negative teacher interactions (D) Negative peer interactions (D) Social comparison (D) Stigmatization (D) Out-of-class school settings (I) Classroom performance (D) Performance evaluation/assessment (D) Within-class grouping/performance levels (I) Performance in group settings (D) Homework situations (D) Other categories social environment
Student	School failure (I) Task mastery (D) Knowledge of dyslexia diagnosis (I) Negative self-concept (D) Negative coping strategies (D) Poor mental/physical health (D) Demotivation (I) Negative emotions (D)
	Other student categories

*D* deductively defined category, *I* inductively defined category

## Results

### Descriptive overview of the included studies

We included 30 studies for the qualitative synthesis. The included studies primarily employed qualitative designs (*n* = 23), while a smaller number used quantitative (*n* = 4) or mixed-methods approaches (*n* = 3). All students whose experiences were described had a diagnosis of dyslexia, a learning disability, or reading difficulties. In many studies, participants reported on their own experiences (*n* = 22), while in others, parents or caregivers (*n* = 11) and teachers/tutors (*n* = 9) provided accounts of children’s or adolescents’ experiences. The reports were based either on current experiences (*n* = 25) and/or retrospective accounts (*n* = 9). Across the included studies, countries most represented were the USA (*n* = 6), UK (*n* = 5), and Australia (*n* = 3); additional studies came from Ireland (*n* = 2), China (*n* = 2), Greece (*n* = 2), the Netherlands (*n* = 2), and Norway (*n* = 2), with single studies from South Africa, Brazil, Spain, Sri Lanka, Slovenia, and New Zealand. A detailed overview of the characteristics of the included is presented in Supplementary Table S2.

### Reported challenges and outcomes of students with dyslexia

**Fig. 2:**
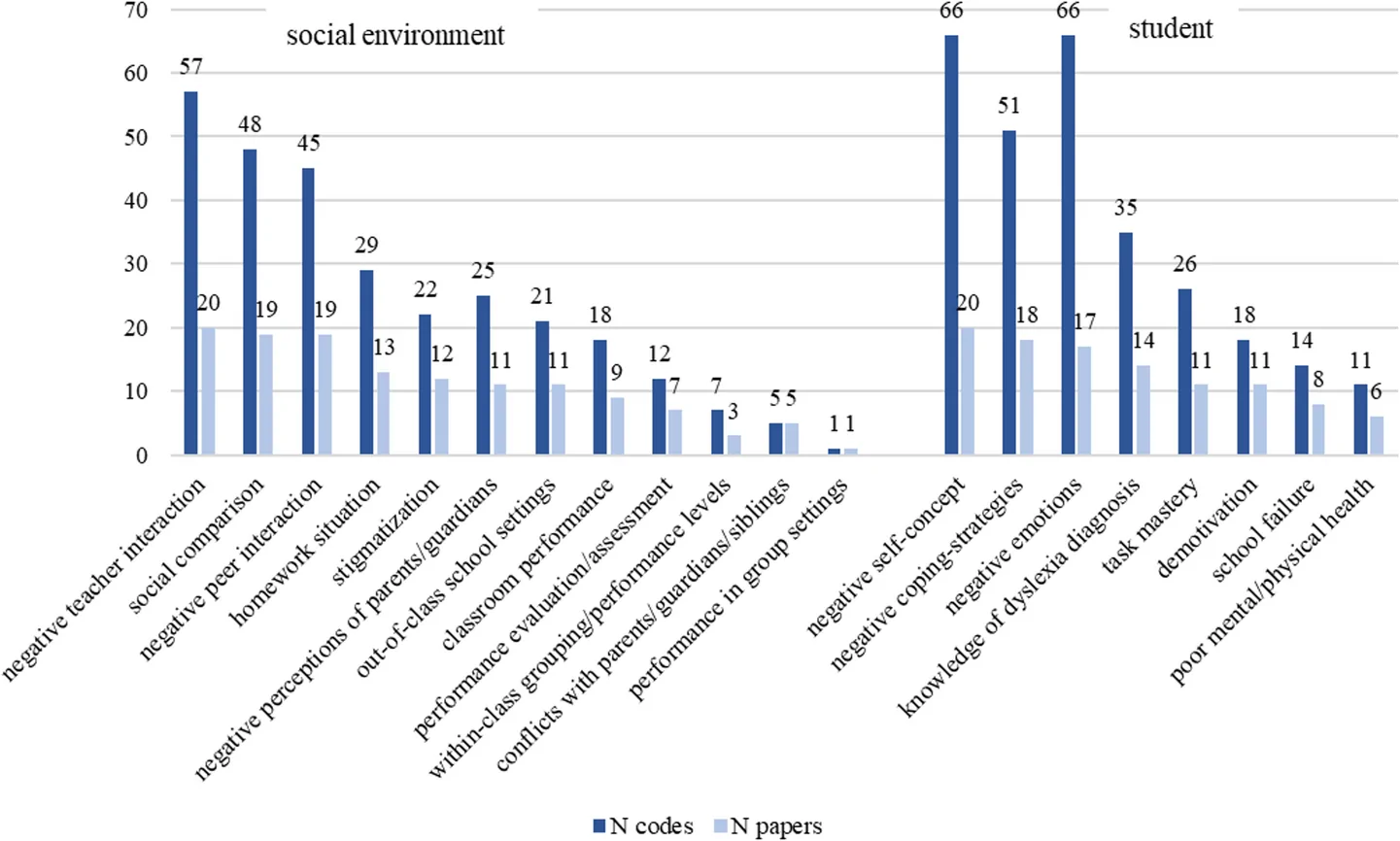
Reported challenges and outcomes faced by students with dyslexia and number of publication

The scoping review identified a wide range of challenging situations experienced by students with dyslexia in a school setting. Through qualitative data analysis, two overarching categories emerged: social environment and student-related stressors (see Fig. 2). Across the included studies, multiple themes were identified reflecting the complex interactions between students, family, teachers, and peers. Within each category, only the most frequently used (in at least 10 studies) codes are further presented in detail. Additionally, we provided an overview of the reported challenges and outcomes across the included studies in Table 4.

**Table 4:** Overview of the reported challenges and outcomes across the included studies

		social environment												student							
		negative perceptions of parents/guardians	conflicts with parents/guardians/siblings	negative teacher interactions	negative peer interactions	social comparison	stigmatisation	out-of-class school settings	classroom performance	performance evaluation/assessment	within-class grouping/performance levels	performance in group settings	homework situations	school failure	task mastery	knowledge of dyslexia diagnosis	negative self-concept	negative coping-strategies	poor mental/physical health	demotivation	negative emotions
1	Alexander-Passe, 2006																	**•**			
2	Casserly & Gildea, 2015			**•**	**•**	**•**	**•**	**•**	**•**								**•**			**•**	
3	Casserly, 2013				**•**	**•**		**•**	**•**				**•**		**•**		**•**	**•**	**•**	**•**	**•**
4	Chan & Mo, 2021	**•**		**•**									**•**								
5	Claassens & Lessing, 2015	**•**	**•**	**•**	**•**	**•**										**•**					**•**
6	Claessen et al., 2020								**•**							**•**	**•**				**•**
7	de Lima et al., 2020					**•**											**•**	**•**	**•**	**•**	**•**
8	Deacon et al., 2020			**•**	**•**	**•**	**•**	**•**	**•**		**•**					**•**	**•**	**•**		**•**	
9	Doikou-Avlidou, 2015	**•**		**•**	**•**	**•**	**•**	**•**					**•**	**•**	**•**	**•**	**•**			**•**	**•**
10	Fernández-Alcántara et al., 2017	**•**			**•**	**•**							**•**				**•**	**•**			
11	Gibson & Kendall, 2010			**•**	**•**	**•**	**•**			**•**	**•**					**•**	**•**			**•**	**•**
12	Hellendoorn & Ruijssenaars, 2000			**•**	**•**	**•**		**•**		**•**						**•**					
13	Jacobs et al., 2022			**•**	**•**	**•**	**•**	**•**	**•**	**•**		**•**			**•**	**•**	**•**	**•**			**•**
14	Leitão et al., 2017			**•**		**•**	**•**	**•**					**•**	**•**	**•**	**•**	**•**	**•**	**•**		**•**
15	Lithari, 2019	**•**		**•**	**•**	**•**	**•**				**•**				**•**	**•**	**•**	**•**		**•**	**•**
16	Lyngsnes & Skjelvåg, 2022			**•**		**•**	**•**						**•**		**•**	**•**	**•**	**•**			
17	McNulty, 2003	**•**	**•**	**•**	**•**	**•**	**•**	**•**	**•**				**•**	**•**		**•**	**•**	**•**			
18	Peries & Indrarathne, 2024	**•**		**•**			**•**												**•**		**•**
19	Rapus-Pavel et al., 2018	**•**	**•**	**•**	**•**				**•**	**•**			**•**	**•**	**•**		**•**	**•**		**•**	**•**
20	Rowan, 2010			**•**				**•**					**•**		**•**	**•**	**•**	**•**			**•**
21	Singer, 2008	**•**		**•**	**•**	**•**							**•**					**•**		**•**	**•**
22	Skaalvik, 2004		**•**		**•**	**•**	**•**		**•**				**•**	**•**	**•**		**•**	**•**	**•**		**•**
23	Stampoltzis & Polychronopoulou, 2009			**•**	**•**	**•**				**•**				**•**		**•**	**•**				
24	Triplett, 2004			**•**	**•**	**•**				**•**											**•**
25	Varghese et al., 2019									**•**											
26	Voorhees, 2006																**•**			**•**	
27	Washburn, 2014	**•**		**•**	**•**			**•**						**•**	**•**			**•**			
28	Wilmot et al., 2023	**•**	**•**	**•**	**•**	**•**	**•**	**•**	**•**				**•**	**•**	**•**		**•**	**•**			**•**
29	Xiao et al., 2022												**•**					**•**	**•**		
30	Zambo, 2004				**•**											**•**	**•**	**•**		**•**	**•**

### Reported challenges in social environment

To enhance readability and avoid redundancy, references in the text are indicated by their assigned numerical identifiers (e.g., 5; 8; 12), which correspond to the references provided in Table 4.

#### Negative teacher interaction

Negative interactions with teachers were characterized by a lack of understanding, insensitive or stigmatizing remarks, and public shaming, often in front of peers. Students reported being labeled as “lazy” or “stupid” due to their struggles with reading and writing (e.g., “You are stupid. It is worse when a teacher says it. It hurts and makes me angry”; 5, p. 33). In another case, a teacher often started reading lessons with: “Let’s listen to A and have a good laugh” (12, p. 233). Such experiences were described as emotionally scarring and degrading. Public humiliation was a recurring theme (*n* = 10 studies [33.33%]; 5; 8; 12; 13; 14; 17; 20; 23; 27; 28). Students reported being asked to read aloud despite their reading difficulties, resulting in feelings of embarrassment and anxiety: “I was told to stand up in front of the class and read out loud… I just couldn’t do it” (8, p. 11). Another participant shared: “My teacher singled me out from the whole class… I felt so awkward and so disappointed” (13, p. 669). The lack of teacher awareness or acceptance of dyslexia was also a major concern (*n* = 9 studies [30%]; 4; 9; 11; 12; 14; 18; 19; 21; 23). Despite having a diagnosis, some students reported that teachers ignored adaptations for oral assessment or refused to reduce homework loads (4; 19). In seven studies [23.33%] (8; 11; 12; 13; 19; 24; 28), the overall school experience was shaped more by these negative teacher–student dynamics than by the learning difficulties themselves.

#### Social compariso

Many students with dyslexia compared themselves negatively to their non-dyslexic peers, often concluding they were less intelligent or capable. Across the studies, comparison with peers was generally described as emotionally distressing and these comparisons contributed to a decline in self-esteem and academic confidence. For example, one participant stated: “There’s a big difference in what I do at school than all the other kids… everyone else in the class is like really good at it except for me” (28, p. 45). Some students tried to avoid situations that would reveal their academic difficulties in front of peers, such as hiding their work or avoiding help-seeking, in an attempt to protect their self-image (21; 22); Others reported sadness and shame upon realizing they were consistently lagging behind their classmates: “I couldn’t cope with the work and couldn’t keep up with my friends” (3, p. 84).

#### Negative peer interaction

Peer relationships were often described as a source of stress and vulnerability for students with dyslexia. Many students experienced social exclusion, teasing, or bullying, often due to their academic struggles. Several studies reported that students with dyslexia felt different, struggled to build or maintain friendships, or were targets of ridicule, particularly when reading aloud or making mistakes in class (8; 17; 19). One participant explained, “When my classmates start laughing I feel embarrassed … If I read in front of the whole class I get nervous, I’m worried that I might mix the letters, and that they will laugh at me” (19, p. 271). Similarly, (28) noted that children feared their difficulties being exposed to classmates and reported anticipatory anxiety around reading tasks. Others expressed a sense of social disconnection, stating they lacked time or confidence to engage in peer activities due to the extra effort schoolwork demanded (13; 22)*.* 

#### Homework situations

The amount of extra time needed to complete homework was frequently emphasized (*n =* 7 studies [23.33%]; 4; 9; 14; 17; 20; 22; 29). This additional workload resulted in feelings of being overwhelmed, as well as in refusal, resistance, and frustration when it came to doing homework (14; 19; 28). It was also associated with parental or self-induced pressure, nervousness, and difficulties concentrating (4; 10; 21). Moreover, long hours of doing homework resulted in limited opportunities for socializing with peers or engaging in extracurricular activities (4; 17; 22).

#### Stigmatization

Many students described feeling “different,” “incapable,” or even “stupid” due to dyslexia. These perceptions that were often reinforced by both teachers and peers (14; 15; 23). These experiences frequently led to internalized shame, reduced academic self-concept, and avoidance of academic or social situations. Several participants reported feeling exposed by dyslexia-related support measures. One student shared, “I think I was the only one in my class who did that so it was seen as a bit odd (…) it was a little uncomfortable to be singled out” (13, p. 674). Others noted that teachers and classmates held misguided assumptions, equating dyslexia with low intelligence or incompetence: “I just thought I was stupid the entire time I was at school” (15, p. 285). Stigmatization also affected help-seeking behavior. Students often avoided asking questions or using learning accommodations for fear of standing out or being labeled. For example, (28, p. 45) described how students found it “embarrassing” to ask for support or use tools that marked them as different. These experiences were compounded by cultural and institutional narratives that framed dyslexia as a deficit, creating barriers to identity development and inclusion (14; 18). Across studies, the effects of stigmatization were consistently associated with negative self-concept. 

#### Negative perceptions of parents/guardians

Parents often struggled to accept and understand their child’s diagnosis, placing considerable pressure on them in the belief that dyslexia could be overcome with rigorous training (*n =* 6 studies [20%]; 4; 5; 9; 10; 15; 19). Some parents expressed skepticism about the need to support their child (15i or did not want their child to receive special treatment (10). As a result, children experienced high levels of pressure and a fear of disappointing their parents or of failing (*n* = 3 studies [10%]; 17; 19; 21). Parents also reported experiencing negative emotions such as sadness and frustration themselves (10). Others described feeling helpless and inadequate in addressing their child’s needs (10; 18), and some perceived their child as a victim due to dyslexia (15).

#### Out-of-class school settings

Out-of-class settings included reading classes, special needs schools, tutoring sessions, and group interventions. Students’ reactions to these environments were mixed, often balancing perceived academic benefits with emotional costs such as social stigma or exclusion. Some students appreciated smaller class sizes, personalized attention, and being grouped with peers who shared similar challenges. This helped reduce pressure and increase feelings of safety (2; 13). For instance, one participant noted: “It was nice to be in a room filled with people who were dyslexic and had the same struggles” (13, p. 673). However, many students expressed discomfort with being pulled out of class. These withdrawals often made them feel different, embarrassed, or excluded. A student reflected: “I don’t like going out of the classroom for extra help” (3, p. 84). Others mentioned being placed in groups that included students with behavioral issues, which contributed to feelings of humiliation and social disconnection (8; 12). One participant described the support class as a “little room” where they felt infantilized, stating, “we’d sit there and count blocks” (8, p. 10). Tutoring outside of school also created varied experiences. While some students valued the individual support and encouragement they received, others found it ineffective or condescending (14; 20). Across the reviewed studies, a common thread was that additional support outside the classroom was only perceived as helpful when it preserved students’ sense of competence and belonging. Otherwise, it could reinforce feelings of inadequacy or marginalization. 

To summarize, students with dyslexia reported a wide range of socially-rooted challenges that shaped their school experience. Negative teacher interactions, including public shaming and lack of support, were particularly harmful. Peer-related issues such as social exclusion and unfavorable comparisons further undermined self-esteem. Homework situations created additional stress. Support in out-of-class settings was helpful when fostering safety and connection but could also lead to embarrassment or further marginalization if not handled sensitively. Overall, these findings reveal that many dyslexia-related difficulties are deeply rooted in social interactions.

### Reported challenges of students

#### Negative self-concept

Students with dyslexia often reported feeling inferior to their classmates, different, or ashamed of their reading or writing difficulties, and described a strong urge to hide their challenges (3; 8; 13; 14; 16; 17; 23; 28). Students mentioned the stigma surrounding dyslexia, which contributed to a negative (reading) self-concept, ultimately affecting their overall identity and self-confidence (9; 10; 13; 15; 22). Self-confidence decreased when mistakes were made (15), leading students to avoid asking questions or actively participating in class (2; 20; 22). Students with dyslexia also attributed their academic struggles and poor grades to personal inadequacy or low intellectual ability (8; 15; 17; 22; 23). This pattern was described as an “attempt by young people with dyslexia to adopt an invisibility approach within the classroom or educational context” (6, p. 59), and aligned with traits of learned helplessness (6). When academic success did occur, it was often attributed to luck or external support rather than one’s own competence (9). Negative self-concept was further reinforced by inappropriate comments from teachers or classmates, teaching styles (13; 15), or experiences of mobbing (14). Students were worried about not being accepted by their classmates and the possibility of not making friends (19), with some reporting that they had very few friends at school (15).

#### Negative emotions

The most frequently reported emotions among students with dyslexia were worry and sadness (*n =* 11 studies [36.67%]; 5; 8; 11; 13; 14; 15; 19; 21; 22; 24; 28), followed by frustration (*n =* 10 studies [33.33%]; 5; 9; 11; 13; 14; 15; 17; 20; 24; 28), anger (*n =* 10 studies [33.33%]; 2; 3; 13; 14; 19; 20; 21; 24; 28; 30), shame (*n =* 9 studies [30%]; 6; 13; 17; 19; 20; 21; 24; 28), and stress (*n =* 6 studies [20%]; 9; 14; 19; 21; 22; 28). Anxiety and fear were reported in five studies [16.67%] (6; 19; 22; 24; 28), followed by annoyance (*n =* 4 studies [13.33%]; 5; 14; 20; 28), nervousness (*n =* 4 studies [13.33%]; 3; 6; 19; 22) and aggression (*n =* 3 studies [10%]; 9; 14; 22). Less frequently mentioned emotions included guilt (*n =* 2 studies [6.67%]; 5; 7), demotivation (*n =* 2 studies [6.67%]; 9; 30), confusion (*n =* 2 studies [6.67%]; 20; 21), loneliness (*n =* 1 study [3.33%]; 17), boredom (*n =* 1 study [3.33%]; 24), and disappointment (*n =* 1 study [3.33%]; 14).

#### Knowledge of dyslexia diagnosis

Students and their families were often already aware of the reading difficulties prior to receiving a formal diagnosis (8; 15; 26). An early diagnosis was sometimes perceived as an internal stigma (8; 13). Negative associations with the diagnosis were linked to prior testing experiences (17) and a general lack of understanding regarding the nature and characteristics of dyslexia. This contributed to self-blame, which in turn had a detrimental impact on students’ self-confidence and learning (*n =* 8 studies [26.67%]; 6; 8; 9; 11; 14; 15; 20; 23). The diagnosis was frequently associated with feelings of being different from peers (14; 20), shame, and a desire to hide their difficulties (6; 12; 16).

#### Task mastery

Students with dyslexia described challenges in initiating, processing, and completing academic tasks; especially those involving reading, writing, and test-taking. These difficulties were often linked to time pressure, cognitive overload, and feelings of inadequacy. A recurring issue was the extended time needed for reading comprehension. One student explained: “I had to read the texts over and over again. I did not grasp the meaning of the text by reading it only one time” (22, p. 111). This repetitive effort led to delays that prevented participation in follow-up class discussions. To cope with these difficulties, some students developed individual strategies such as highlighting text elements with markers of different colors or relying on assistive technology (9; 16). However, even when strategies were applied, the success of task completion often depended on external support and teacher understanding. For example, some teachers failed to provide sufficient time or adapt tasks to their needs: “The teachers dictated too fast. He [my son] didn’t manage to write everything” (19, p. 271).

#### Demotivation

Students reported not enjoying school (7), associated with persistent feelings of inferiority in comparison to their classmates (2). Students’ demotivation was further linked to teaching practices perceived as humiliating, such as being asked to read aloud in front of the class, frequent comments about reading and writing difficulties, and discussions about dyslexia-related challenges (9; 15; 30). Both students and their parents described feelings of being ‘unable to learn’, recurrent experiences of failure, a gap between performance and expectations, and patterns of task avoidance (2; 20; 26). 

To summarize, students with dyslexia experience a range of interrelated emotional, cognitive, and social challenges that significantly affect their school experience. A pervasive theme across studies was a negative self-concept, often rooted in feelings of inferiority, shame, and a desire to remain invisible in the classroom. Many students adopted avoidant coping strategies and reported strong negative emotions such as worry, frustration, and anger. The diagnosis itself was linked to mixed feelings, including relief but also self-blame and a sense of being different. Students also reported substantial difficulties in mastering academic tasks due to time constraints, cognitive overload, and insufficient support, which further reinforced demotivation and feelings of failure. Together, these findings draw attention to the concrete situations that students with dyslexia experience as particularly challenging, offering important insights for developing more supportive and inclusive educational environments.

## Discussion

This scoping review aimed to systematically identify and categorize the school-related situations that students with dyslexia experience as particularly challenging. Drawing on 30 peer-reviewed studies, the findings show that while dyslexia is commonly framed solely in terms of reading and writing difficulties, its effects deeply permeate social and emotional aspects of school life. Especially the first-person accounts in the included qualitative studies reveal deeply personal and emotionally charged school-related experiences. Therefore, by classifying the challenges into two main thematic categories—social environment and student-related stressors—this review advances our understanding of the lived experiences of students with dyslexia. Interpreted through bioecological and person–environment fit lenses, the themes identified here point to multi-level design changes rather than solely individual remediation. Many distressing experiences arise not from dyslexia per se but from how routines make difficulties public, constrain autonomy, or signal low expectations—mechanisms that can also affect other struggling readers. In this sense, the findings highlight the need to design school systems that proactively minimize barriers and emotional risk factors rather than reactively addressing distress once it occurs.

### Impact of social environment

This category encompassed negative teacher and peer interactions, social comparison, homework situations, stigmatization, negative perceptions of parents/guardians, and out-of-class school settings. A central insight from the review is how routine classroom practices often intensify emotional distress. Reading aloud was frequently described as distressing, with students reporting feelings of humiliation and anxiety (Hellendoorn & Ruijssenaars, 2000; Jacobs et al., 2022). Social comparisons further reinforced a sense of inferiority, as students often described being slower than peers or struggling to keep up (Wilmot et al., 2023). Negative peer interactions, such as teasing or exclusion, were commonly reported during reading tasks, leading to embarrassment and social withdrawal (Rapus-Pavel et al., 2018). Homework situations added stress, with long durations limiting students’ free time and contributing to frustration (Xiao et al., 2022). Some students also felt stigmatized by special accommodations or being pulled out of class, which made them feel different (Jacobs et al., 2022).

Such classroom and home routines can amplify socio-emotional strain and frustrate students’ need for relatedness (Schmidt et al., 2020). To counteract these risks, systemic approaches such as Universal Design for Learning (Zhang et al., 2024) can reduce the visibility of individual differences by offering multiple means of engagement, representation, and expression for all learners. Embedding socio-emotional learning into daily instruction, shifting comparison from peer ranking to personal progress, adding brief peer-awareness activities about diverse learning profiles, using strengths-based language for dyslexia, ensuring access to a trusted adult/mentor to support identity and belonging, and giving the “right-size” homework with different format choices (e.g., listen-then-respond; chunked tasks) can be some of the strategies to address the identified categories. At a school policy level, such adjustments should be embedded within whole-school frameworks (e.g., Mathie & Wals, 2022) that align curriculum design, assessment practices, and learning environments with emotional safety and equitable participation.

### Impact of student-related stressors

This category included negative self-concept, negative coping strategies, negative emotions, knowledge of dyslexia diagnosis, task mastery, and demotivation. Many students developed a negative self-concept, often feeling “stupid” or “less capable” than their peers (Lithari, 2019). These internal beliefs led to behaviors like avoiding participation or help-seeking (Claessen et al., 2020). Students also adopted avoidant coping strategies, such as memorizing texts or pretending to be ill, to escape reading-related tasks (Casserly, 2013; Lyngsnes & Skjelvåg, 2022). Emotional responses like sadness, shame, and anger were prevalent, particularly following public mistakes or criticism (McNulty, 2003; Casserly, 2013). These student-reported beliefs and emotions are consistent with a transactional account in which repeated school experiences shape self-views and perceived school belonging over time (Chen et al., 2025).

The dyslexia diagnosis itself was linked to mixed feelings—some felt relief, others felt labelled and different (Leitão et al., 2017). Diagnostic labels can be both enabling and burdensome (Werkhoven et al., 2022). When explained in strengths-based language and paired with positive framing, peer awareness, and mentorship, labels can normalize help-seeking and reduce uncertainty (Abbey & Valsiner, 2005; Blajeski et al., 2022; Sims et al., 2021). When framed narrowly as deficits, they can signal low expectations and invite stigma (Link & Phelan, 2001; Riddick, 2000). Difficulties with task mastery, especially under time pressure, contributed to demotivation and reduced engagement in learning (Doikou-Avlidou, 2015; de Lima et al., 2020). In this category, student narratives were central, and there was relatively limited inclusion of teacher, parent, or peer viewpoints. Future interventions could address these dynamics through teacher-led reflective dialogues on mindset and labeling, peer mentorship programs that emphasize diverse learning pathways, and school-based mentoring models aimed at fostering academic resilience and positive self-concept. Integrating socio-emotional and self-efficacy training into literacy instruction may also help reduce avoidance behaviors and reinforce students’ sense of competence and belonging.

### Implications for research and educational practice

The aim of this review was not only to describe specific challenges of students with dyslexia, but also to highlight how they emerge and can be exacerbated within the school environment. The findings suggest that the school experiences of students with dyslexia are profoundly shaped by interpersonal dynamics and systemic school routines—far beyond the well-documented academic challenges. It seems that many of the most distressing experiences are not a direct effect of difficulties in reading and writing, but rather of how school settings can enhance negative emotions—for example, by exposing students’ learning difficulties to the entire class (Doikou-Avlidou, 2015; McNulty, 2003; Skaalvik, 2004). Notably, practices identified as distressing for students with dyslexia are likely also harmful for other students who struggle with reading, spelling, and writing who lack a formal diagnosis. Thus, the practical implications extend beyond dyslexia to broader literacy support and classroom culture.

The review identifies a critical mismatch between the emotional needs of students with dyslexia and prevailing school practices. Despite increasing awareness and more frequent diagnosis, many schools continue to uphold educational practices that may unintentionally marginalize or distress students with dyslexia. This insight calls for a shift from individual-level interventions toward school-wide change to create more inclusive and emotionally safe environments. Frameworks such as whole-school approaches (Oberle et al., 2016) can help ensure that inclusive practices are not confined to individual classrooms but embedded across policy, curriculum, and culture. Schools can mitigate risk by co-constructing student-approved explanations of dyslexia, using private rather than public feedback, and ensuring access to a trusted adult/mentor to support identity and belonging (Deacon et al., 2020; Gibson & Kendall, 2010). Teacher training that places greater emphasis on socio-emotional literacy, stigma reduction, and inclusive pedagogies—ensuring that educators are equipped to respond to the socio-emotional as well as academic needs of their students—is critical for sustaining this shift. Pre- and in-service teacher education should include training in science-based reading acquisition and intervention strategies (e.g., Structured Literacy).

Addressing the person–environment misfit is essential for ensuring that students with dyslexia and/or other learning difficulties receive not only appropriate academic support but also the socio-emotional scaffolding they need to thrive. Multi-tiered systems of support (Vetter et al., 2024) that integrate academic, social, and emotional screening may be useful in ensuring that early signs of distress are identified before they escalate. Future longitudinal studies could offer valuable insights into how changes in school culture and practices would influence both academic outcomes and emotional well-being of students with dyslexia over time. Moreover, greater attention should be given to the voices of students themselves, as their perspectives are key to understanding the support they need and to developing responsive, inclusive, and emotionally supportive school systems.

### Limitations

The scoping review process comes with several limitations. First, the findings are based on studies stemming from different contexts and school systems, thereby reducing the generalizability of our findings. Teacher training, diagnostic protocols, classroom practices, and support systems differ considerably across countries. Accordingly, the nature of the challenges described and the resources available to address them also vary significantly. This cross-country variability is expected within a bioecological perspective, where school procedures and culture shape how similar difficulties are experienced and managed (Rosa & Tudge, 2013). Second, the review captures subjective and heterogeneous experiences. Challenges are highly individual and influenced by a wide range of interacting factors—including student characteristics, severity of dyslexia, family support, friendships, teacher attitudes, and school culture. A situation perceived as distressing by one student may be experienced as manageable by another. This highlights the need for caution in generalizing across all students with dyslexia. Third, the review includes studies with participants described as having dyslexia, reading and/or writing difficulties—often without reporting standardized diagnostic criteria, reflecting the broader issue of inconsistent definitions and assessments of dyslexia across studies as well as countries. This variability in the studied population might affect the specificity of our conclusions.

## Conclusion

Given the prevalence of emotionally distressing situations linked to routine school practices, a key implication of this review is the need to reframe inclusion beyond academic accommodations. While extended time on exams or assistive technologies are essential, they are insufficient if the broader socio-emotional climate in schools remains exclusionary or stigmatizing. Many of the reviewed studies described how students with dyslexia reported feeling different, less intelligent, or embarrassed in everyday school interactions (e.g., Casserly, 2013; Deacon et al., 2020; Jacobs et al., 2022; Leitão et al., 2017). Emotional distress was often triggered not by the diagnosis itself, but by educational practices that undermine students’ confidence and reinforce internalized stigma (Deacon et al., 2020; McNulty, 2003). This calls for structural and relational changes that prioritize emotional safety, positive identity development, and a sense of belonging. Educators must critically reflect on routines that may cause disproportionate distress for students with dyslexia. Alternative practices such as recognizing strengths and areas of competence, offering private instead of public feedback, recognizing individual progress, creating dyslexia-informed classroom cultures, and flexible participation options could significantly improve the daily school experience of students with dyslexia. It might also protect them from the cumulative impact of daily micro-exclusions such as the ones described above (Elliot et al., 2007; Lise et al., 2025; MacKay, 2004). Ultimately, inclusive education must be more than a set of accommodations—it must be a culture shift. Schools that prioritize relationships, recognize diversity in learning, and attend to the emotional dimensions of education will be better equipped to support all students, including those with dyslexia.

## Supplementary Information

Below is the link to the electronic supplementary material.

Supplementary Material 1 (DOCX 37.6 KB)

Supplementary Material 2 (DOCX 47.8 KB)
