# Supplementary material for: Challenging school-related situations faced by students with dyslexia: a scoping review
Source: Ann Dyslexia. 2025 Dec 12;76(3):587–611. doi: 10.1007/s11881-025-00356-9 (PMC13759582; doi:10.1007/s11881-025-00356-9)
Supplement: Supplementary file 1 [file 11881_2025_356_MOESM1_ESM.docx]

**Supplementary Material**

**Table S1** Coding manual

| Main category | Subcategory | Description of category | Examples |
| --- | --- | --- | --- |
| Social environment | Negative perceptions of parents/guardians (ded.) | Like teachers, parents are among the ‘significant others. Their perception of school challenges faced by their child has a major impact. Some parents pressure their child, have unrealistic expectations or accuse them of being lazy. | “There’s very few parents who see dyslexia as a challenge to be overcome. Whereas many of them will either see it as … as their child is a victim, and therefore school is bad or they will see dyslexia as making their child special and so […] normal rules don’t apply.”  (Lithari, 2019, S. 289) |
|  | Conflicts with parents/guardians/siblings (ded.) | Conflicts or insults may arise between the student with dyslexia and their parents/siblings. Siblings may feel that they are not receiving enough attention. | “My parents do not know what is going on in my life … they only say that I am not learning; They only shout at me.”  (Classens & Lessing, 2015, S. 33) |
|  | Negative teacher interactions (ded.) | Negative teacher behavior includes addressing students inappropriately, making negative remarks or discouraging comments and making specific statements about reading (e.g., statements that worry students with dyslexia or trigger negative feelings). It also includes teachers getting angry, when students ask for help, having no understanding of the diagnosis, or not knowing how to deal with it. When students read, the teacher treats them like toddlers or beginners. Students rarely get praised, as only good performance (reading and writing) in the main subjects counts. | “Comparatively, a lack of knowledge or belief about dyslexia, and an inability to provide adequate support were common amongst the participants who reported negative experiences with educators. This is captured in one participant’s reflection of school. I would have been much happier if all my teachers from my first school weren’t so mean and discouraging, and then I got a bad teacher... more of a mean teacher...she started to highlight how I kept failing everything... teachers always being mean to me, they didn’t believe in dyslexia, so they sort of treated me more hard.” (Leitão et al., 2017, S. 328) |
|  | Negative peer interactions (ded.) | Peers behave negatively towards students with dyslexia, for example by laughing at them or bullying them. | “Guys in the class who think they are funny and make remarks … like you are dumb, why don’t you understand, it’s so straight forward? Then it makes me very negative … then they think you are only stupid … that was not nice … it makes me angry and aggressive.” (Classens & Lessing, 2015, S. 33) |
|  | Social comparison (ded.) | Children compare themselves and their performance with others. These comparisons mostly reveal inferior performances. | “For example, Emma compared her work to that of others in her year with negative outcomes for her self-perception: They’re really good at something and I’m comparing myself to them and I’m thinking ‘Oh, I’m rubbish at that and everything’.” (Lithari, 2019, S. 288) |
|  | Stigmatization (ded.) | Stigmatization can arise from negative expectations based on the dyslexia label. For example, being seen as stupid and not able to read, being given different tasks and attention, being taken out of lessons and not being given opportunities to use aids. | “My parents were told that my brother and I, and my brother is also dyslexic, were too stupid and we needed to go to a special needs school. (Josephine, 41). The narratives reveal that some participants felt ‘stupid’ because of difficulties they experienced at school and this type of narrative came from most participants, highlighting stigmatization: So I just thought I was stupid, I thought I was stupid the entire time I was at school.”  (Lithari, 2019, S. 285) |
|  | Out-of-class school setting (ind.) | Challenges of taking students with dyslexia out of class to support them in other settings (e.g., individual settings or learning support classes). | “Similar experiences can be seen at secondary school as Martha (21) recounts her feelings about being moved to a special needs class: It was a ‘little room’ … while everyone else would do their maths lesson, and then I’d go into this support group and we’d sit there and count blocks. I was with the kids that were deemed naughty or kids who were seen to have special needs and things.” (Deacon et al., 2020, S. 10) |
|  | Classroom performance (ded.) | Classroom performance includes “standard situations” such as reading aloud, writing on the board, responding to questions from the teacher when you don't know the answer, being encouraged to "speed up or improve" and limited time on exams. | “However, 15 children were intimidated when asked to speak in front of their peers for fear of making mistakes, while 10 children were still fearful before participating in such.”  (Casserly, 2013, S. 86) |
|  | Performance evaluation/assessment | Challenges resulting from performance assessment, such as practicing spelling but still getting bad grades for dictations or practicing reading and still struggling, and still reading worse than others. Students with dyslexia getting bad grades. | “One teacher doesn’t understand my problems. If you get a bad grade, she accuses you of not studying ... I think it’s because no one has told her anything. I would like her to understand me when I have oral assessment. In tests I have adaptations, but in the oral assessment she doesn’t understand.”  (Rapus-Pavel et al., 2018, S. 271) |
|  | Within-class grouping/performance levels (ind.) | Occurring challenges due to performance levels. | “When I went there [secondary education], I was placed in the bottom division for maths and English. But they put me in the top division for the sciences and art […] and music. […] It was really disorientating, actually […] because I was never quite sure what was expected of me, you know, one minute they seemed to be expecting me to be intelligent and ok and the next minute they seem to think that I was really stupid and didn’t know how to do things and […] it also meant that I was always being put with different students, so it was difficult to make friends. And that was another thing, which I found quite difficult.”  (Lithari, 2019, S. 284) |
|  | Performance in group settings (ded.) | Challenges that arise from performing in group settings. | “Being involved in a group intervention meant that attention was drawn to her difficulties, and led to further pressure to learn, which amplified the experience of stress, embarrassment, and frustration. She explained that she desired to take ownership of her learning without the risk of feeling judged by the group or by the support facilitators.”  (Jacobs et al., 2022, S. 673) |
|  | Homework situations (ded.) | Students with dyslexia need more time for homework, work more slowly and have no motivation/show frustration doing it. They use avoidance strategies and have no time for leisure activities or friends. | “Our results confirm that dyslexia extends the time it takes children to complete homework.” (Xiao et al., 2021, S. 499) |
|  | Other categories social environment |  |  |
| Student | School failure (ind.) | This category includes any kind of school failure, fear of school failure or actual school failure. Not reaching one’s potential due to a lack of education. | „He’s afraid that he won’t be able to do it, that he will be get a low grade. He says ‘Oh no, what if I get a grade of two [satisfactory]’. He’s afraid of the grade ... He’s afraid of failure.”  (Rapus-Pavel, 2018, S. 270) |
|  | Task mastery (ded.) | Written tasks are difficult to understand due to reading difficulties. For example, some math tasks cannot be solved because instruction is not understood. The same applies for other subjects, particularly those that are text-heavy. Slow reading means that much more time is needed to complete tasks, particularly those involving texts. | “Josephine was never confident while she attended school and George had problems when he could not understand lessons: Sometimes in maths, ‘cause … I really don’t get it and feel like I can’t do it and break down. So … this happens from there sometimes if … I don’t get it and break down.” (Lithari, 2019, S. 290) |
|  | Knowledge of dyslexia diagnosis (ind.) | The effect of (not) knowing the dyslexia diagnosis. | “I was diagnosed in primary school but no one bothered to tell me. […] It wasn’t until I went to senior school. So you thought it was your fault and it was your … I just thought I was an idiot. […] And I was diagnosed quite early but, um, I don’t know, the school never bothered to point anything out like that to me.”  (Lithari, 2019, S. 290) |
|  | Negative self-concept (ded.) | Negative conclusions are drawn from everyday school life and interaction with others. Students with dyslexia may consider themselves to be stupid, inferior or not accepted from others. | “So I just thought I was stupid, I thought I was stupid the entire time I was at school.”  (Lithari, 2019, S. 285) |
|  | Negative coping-strategies (ded.) | Negative coping strategies include coping with demands, failure, bad grades, pressure and negative feelings (e.g., avoidance strategies when reading such as withdrawing or becoming aggressive). | “These feelings were related to the adoption of coping strategies such as avoidance and to problems in participating in play with others. He does not participate in competitions or anything because he says no ... that he won’t be able. He has no physical problem, is able to do anything, but ....” (Fernándet-Alcántara et al., 2017, S. 11) |
|  | Poor mental/physical health (ded.) | This category includes symptoms of stress, depression, anxiety, suicidal thoughts, sleeping difficulties, tiredness and somatic complaints, such as headaches and abdominal pain. | “My stomach was in knots when I knew that I had to read and it still is in a situation where I have to read aloud. My heart was beating and I began to sweat. I stuttered and everything was cluttered up.” (Skaalvik, 2004, S. 116-117) |
|  | Demotivation (ind.) | Demotivation can originate from various sources, such as poor performance or interaction with others. | “There was a ‘resignation’ of being unable to move to a higher set: this, it seemed, impacted upon self-esteem and motivation.”  (Gibson & Kendall, 2010, S. 189) |
|  | Negative emotions (ded.) | Negative feelings which are directly related to reading (performance). These include anger, fear, aggression, shame, frustration and sadness. | “Kimberly discussed dyslexic learners in general and how they feel about their dyslexia: I think it [dyslexia] does upset them. And sometimes you get kids who just become very tearful when they think about it. But usually they will avoid thinking about it as much as possible.”  (Lithari, 2019, S. 289) |
|  | Other categories student |  |  |
