# Supplementary material for: Challenging school-related situations faced by students with dyslexia: a scoping review
Source: Ann Dyslexia. 2025 Dec 12;76(3):587–611. doi: 10.1007/s11881-025-00356-9 (PMC13759582; doi:10.1007/s11881-025-00356-9)
Supplement: Supplementary file 2 [file 11881_2025_356_MOESM2_ESM.docx]

**Supplementary material**

**Table S2** Overview of the included studies

| # | Author(s) and year | Study type | Study design | Sample | Country | Type of reporting | Reported situations |
| --- | --- | --- | --- | --- | --- | --- | --- |
| 1 | Alexander-Passe, 2006 | Quantitative | Cross-sectional | 19 children and adolescents with dyslexia (7 female, 12 male)  Age: 10-17 years | UK | Self-report of current situation | - Gender-specific differences in problem solving; - Female students with dyslexia are more likely to adopt emotion-focused, avoidant coping strategies and social distraction; - Male students with dyslexia tend to use task-oriented coping strategies |
| 2 | Casserly & Gildea, 2015 | Mixed methods | Longitudinal | 16 children with dyslexia  Age: 11-12 years  14 teachers | Ireland | Self-report and teacher report of current situation | - Separative setting has a beneficial effect on self-esteem, as reading takes place separately and without stigmatization in a protected environment |
| 3 | Casserly, 2013 | Mixed methods | Longitudinal | 10 reading schools/classes (3rd/4th class), 8 reading class teachers, 2 reading school teachers, 20 sets of parents, 20 children, 20 mainstream class teachers, 20 support teachers working in mainstream schools | Ireland | Self-report and teacher report of current situation | - Social comparison, being slower than others; - Being exposed to remedial lessons outside the classroom; - Report of avoidance strategies (e.g., sharpening pencil, feigning stomach ache or tiredness, going to the toilet etc.); - Low self-esteem after special support; - Psychosocial symptoms such as difficulty sleeping, nausea before exams, social withdrawal and depression |
| 4 | Chan & Mo, 2021 | Qualitative | Semi-structured interviews | 20 parents (2 fathers and 18 mothers) of children with diagnosed dyslexia or diagnosed combined dyslexia and ADHD  Age: 7-12 years | China | Parent report of current situation | - Individual factors causing parental stress: characteristics of children and study problems for children; - Family factors causing parental stress: daily responsibilities and maternal role inequalities; - Social and cultural factors causing parental stress: social comparisons and promoting a blaming culture; - School factors causing parental stress: educator’s power and control; - Coping methods used by parents |
| 5 | Claassens & Lessing, 2015 | Qualitative | Semi-structured interviews | 6 young adults (3 female, 3 male)  Age: 18-21 years | South Africa | Self-report of retrospective situation | - Social comparisons, impatience, and stigmatization by teachers |
| 6 | Claessen et al., 2020 | Qualitative | Semi-structured interviews | 20 educators working with young people with dyslexia  Age: 5-18 | Australia | Teacher report of current situation | - Defining dyslexia; - Approach to educational support; - Educators’ perceptions of how young people respond to diagnosis; - Family context; - Educational context; - Perceived mental health implications |
| 7 | de Lima et al., 2020 | Quantitative | Cross-sectional | 61 students (62% male), including 31 students (71% male) diagnosed with dyslexia  Age: 7-14 years | Brazil | Self-report and parent report of current situation | - Higher scores in anxiety, depression, withdrawal, and externalizing behaviors such as aggression; - High scores in negative self-evaluation, guilt, suicidal thoughts, feeling concern, performance comparison, sleeping difficulties, fatigue, and problems in interacting with peers at school |
| 8 | Deacon et al., 2020 | Mixed  methods | Cross-sectional | 442 participants 15 adults (9 female, 6 male)  Age: 23-64 years | UK | Self-report of retrospective and current situation | - Insults from teachers such as "are you stupid?", "you’re thick"; - Making mistakes and letting them drag you down; - Being laughed at by teachers, feeling deficient or limited in own abilities; - Teacher influence in subject selection; - Slower task completion compared to classmates, feeling deficient; - Problems after diagnosis, feeling stigmatized and excluded by classmates; - "Direct" bullying by classmates and "indirect" bullying by teachers; - Reading aloud in front of the class; - Physical violence by classmates; - No time or space for assistive technology that is perceived as helpful |
| 9 | Doikou-Avlidou, 2015 | Qualitative | Semi-structured interviews | 10 students (7 male, 3 female)  Age: 18-27 years | Greece | Self-report of retrospective and current situation | - Effects of teacher behavior, parental behavior, and peer behavior (especially negative behavior) on self-worth, academic trajectory, social skills, and relationships; - A retrospective view on school situations, teachers, homework context, resilience factors, and university studies |
| 10 | Fernández-Alcántara et al., 2017 | Qualitative | Focus-group open-ended interviews | Focus-group dyslexia: 9 parents (5 mothers, 4 fathers) of 6 children  Age: 12 years | Spain | Parent report of current situation | - Parenting emotions: feelings of helplessness; - Daily experiences: concerns about the treatment their children received at school and negative attitudes toward special allowances (e.g., being less strict with spelling errors, giving extra time to complete tasks or exams); - Social relationships: comparisons with peers, low self-esteem, shame; - Concerns about the future |
| 11 | Gibson & Kendall, 2010 | Qualitative | Semi-structured interviews | 5 university students | UK | Self-report of retrospective situation | - Differentiated teaching practices, negative teacher attitudes, lack of support, low teacher expectations; - Verbal abuse within their peer groups but no physical bullying (e.g., being laughed at), negative feelings triggered by social comparison during reading |
| 12 | Hellendoorn & Ruijssenaars, 2000 | Qualitative | Semi-structured interviews | 27 adults (15 female, 12 male) with dyslexia  Age: 20-39 years | Netherlands | Self-report of retrospective and current situation | - Significant impact of dyslexia on daily life; - Even though individuals affected by dyslexia learn to cope with it in adulthood, the negative memories and experiences from their school years often persist; - Negative performance evaluations by teachers contribute substantially to the development of a negative self-image; - Teachers frequently judged students (e.g., calling "stupid," "lazy," etc.), often due to a lack of knowledge and acceptance of dyslexia; - Affected students are laughed at and excluded by peers and experience humiliation by teachers in front of the entire class |
| 13 | Jacobs et al., 2022 | Qualitative | Semi-structured interviews | 14 university students (11 female, 3 male)  Age:18-36 years | UK | Self-report of current situation | - Managing feelings of inferiority: stigmatization, a lack of understanding of dyslexia among peers and educators; - Adapting to the learning environment: limited accommodations; - Experience of learning support: limited learning support |
| 14 | Leitão et al., 2017 | Qualitative | Semi-structured interviews | 13 children with dyslexia (7 female, 6 male)  Age: 10-16 years  21 parents (19 mothers, 2 fathers) | Australia | Self-report and parent report of current situation | - Diagnosis plays a decisive role, the diagnosis helped to categorize the problem; - Before the diagnosis, children realized that they could answer questions that were asked verbally, while they could not understand and answer written questions. They then considered themselves to be stupid and different from their peers; - Teachers emphasized the linguistic inability in the classroom and perceived dyslexia as an excuse |
| 15 | Lithari, 2019 | Qualitative | Semi-structured interviews | 6 children (4 male, 2 female) with dyslexia aged 12-13 years, parents of the younger participants, 4 young adults (3 male, 1 female) with dyslexia aged 17-26 years, 4 adults (2 male, 2 female) with dyslexia aged 41-56, one education professional (46 years) | UK | Self-report (children, young adults, adults), teacher report, and parent report of retrospective and current situation | - The stigma experienced was a perceived lack of intelligence, which disappeared after a dyslexia diagnosis; - Diagnosis is a crucial part of identity development; - Participants often had to repair their sense of self; - Teacher behavior plays a prominent role in the learning of children with dyslexia; - Negative teacher behavior leads to anxiety, fear of failure, and contributes to poor self-esteem |
| 16 | Lyngsnes & Skjelvåg, 2022 | Qualitative | Semi-structured interviews | 4 secondary school students (1 male, 3 female) diagnosed with dyslexia  Age: 14-16 years | Norway | Self-report of current situation | - Personal coping strategies related to schoolwork; - Foreign language torment; - Emotional impact; - Struggling with educational support: the significance of getting an early diagnosis to provide relevant support at an early stage, the necessity of teacher competence |
| 17 | McNulty, 2003 | Qualitative | Narrative biographical interviews | 12 adults (4 female, 8 male) with dyslexia  Age: 25-45 years | USA | Self-report of current situation | - Reading aloud in front of the class is described as a particularly traumatizing experience; - Situations of failure at school are described as shameful and humiliating; - Repeated situations (e.g., being called to read aloud every week) resulted in heightened emotional insecurity and self-doubt |
| 18 | Peries & Indrarathne, 2024 | Qualitative | Individual and focus-group semi-structured interviews | 30 caregivers of children with dyslexia | Sri Lanka | Parent report of current situation | - Lack of knowledge among primary caregivers; - Primary caregivers’ challenges in raising a child with a learning disability; - Primary caregivers’ demand for more resources |
| 19 | Rapus-Pavel et al., 2018 | Qualitative | Semi-structured interviews (open interviews) | 4 adolescents (1 female, 3 male) with dyslexia, their mothers and teachers  Age: 12-13 years | Slovenia | Self-report, teacher report, parent report (mothers) of current situation | - A comparison of the perspectives of adolescents, mothers, and teachers shows the different ways of seeing and dealing with dyslexia; - Stressful situations emerged with regard to learning activities and assessment, teachers’ lack of understanding, rejection by classmates, and mothers’ high expectations; - This leads to expectations for support services: More understanding for emotional stress in connection with dyslexia, more adjustments in the classroom |
| 20 | Rowan, 2010 | Qualitative | Semi-structured interviews | 4 university students with dyslexia  Age: not specified | New Zealand | Self-report of current situation | - Students experienced teacher and system ignorance of their learning diﬃculties and needs; - Inappropriate support oﬀered in an ad hoc manner impacted their success: Many of the support measures were perceived as humiliating and belittling ; - Students perceived their learning with dyslexia as diﬀerent from their peers and creating diﬃculties within the existing educational system |
| 21 | Singer, 2008 | Qualitative | Semi-structured interviews | 60 students (24 female, 36 male)  Age: 9-12 years | Netherlands | Self-report of current situation | - Difficult situations: entrance tests, dictations, reading aloud in class, math and history tests that depended on reading skills; - The students relied most strongly on support from their parents; - Teachers and peers were more likely to be seen as threats to self-esteem |
| 22 | Skaalvik, 2004 | Qualitative | Semi-structured interviews | 3 adults (2 female, 1 male) with dyslexia  Age: 30-47 years | Norway | Self-report of retrospective situation | - Various indications of negative affect (e.g., experienced anxiety in situations related to reading or where “weaknesses” become visible) - Using self-defensive strategies to hide reading and writing problems; - Feelings of being perceived negatively by their classmates |
| 23 | Stampoltzis & Polychronopoulou, 2009 | Qualitative | Semi-structured interviews | 16 university students (5 female, 11 male) with dyslexia  Age: 19-26 years | Greece | Self-report of retrospective situation | - Social comparison, resulting in the feeling of being inferior, misunderstood, and stupid; - Lack of understanding and pressure from teachers, especially in elementary school, with a more positive experience in secondary school; - Peer situations during breaks and group work; - Good friendships made school situations bearable; - Increased security through diagnosis |
| 24 | Triplett, 2004 | Qualitative | Single-case study;  Written retrospective narratives, open-ended interviews | 1 child with dyslexia (male, Grade 6) | USA | Self-report, parent report (mothers), teacher report (reading assistant) of retrospective and current situation | - Concerns about the social comparisons; - Interactions with the teacher were associated with many, sometimes diffuse feelings, because the pupil was unsure about what the teacher wanted from him; - Teacher felt insecure when dealing with the child with dyslexia and hindered in teaching; - Teacher’s professional impact was called into question by the pupil, making the relationship difficult |
| 25 | Varghese et al., 2019 | Quantitative | Longitudinal;  Intervention (randomized controlled trial) | 503 children (287 in kindergarten, 216 Grade 1), 51 teachers | USA | Teacher report of current situation | - Conflictual teacher–child relationship were negatively associated with children’s literacy achievement and social competencies |
| 26 | Voorhees, 2006 | Qualitative | Retrospective narrative about the literacy life of one adolescent | 1 teacher (female) | USA | Teacher report of retrospective situation | - Classroom challenges in engaging struggling adolescent readers; - Misplacement of students; - Lack of tailored literacy instruction; - Emotional struggles; - Reflections on systemic educational shortcomings |
| 27 | Washburn, 2014 | Qualitative | Semi-structured interviews, analysis of artefacts, documents | 4 mothers of 5 children with dyslexia  Age: 8-12 years | USA | Parent report (mothers) of current situation | - Mothers identified literacy difficulties (investigator role), advocated for support (advocate role), and provided academic help (tutor role) to their children; - Situations in schools that have a negative impact on the self-esteem of children with dyslexia (e.g., situations in which the child is laughed at in the presence of the teacher if he/she does not read well); - Lack of school-home partnerships |
| 28 | Wilmot et al., 2023 | Qualitative | Semi-structured interviews | 17 children with dyslexia and their mothers  Age: 9-14 years | Australia | Self-report, parent report (mothers) of current situation | - Self-esteem, stigma, and shame related to “difference” as well as the protective function of parent support and the language of neurodiversity; - Children’s school-based emotions, emotion regulation, and mother’s perceptions of meltdowns, school refusal, and homework resistance as signs of exhaustion and overwhelm; - Perceptions of school as a poor “person-environment fit” for children with dyslexia, school connectedness, and peer relationships |
| 29 | Xiao et al., 2022 | Quantitative | Cross-sectional study | 3993 students (2076 male, 1803 female) of which 114 were diagnosed with dyslexia (80 male, 34 female)  Grade 2-6 | China | Parent report of current situation | - Children with dyslexia spent significantly more time on homework and had higher scores for depression and stress; - Dyslexia had a direct link with depressive symptoms; - Time spent on homework and stress could mediate the association between dyslexia and anxiety/depressive symptoms |
| 30 | Zambo, 2004 | Qualitative | Semi-structured interviews with children and teachers, field notes, diary entries | 6 teachers (5 female, 1 male), 11 children (one female, 10 male)  Grade 5-9 | USA | Self-report and teacher report of current situation | - Situations at school in which children are called stupid and lazy by other children; - In many situations, strenuous reading is avoided and this also reduces the opportunities to practise; - Some of the students used defiance to cope with their experiences, to hide the hurt they were feeling inside |
